# MRI evaluation of cranial pathologies in rapidly progressive early puberty cases aged 8-9

**DOI:** 10.3389/fendo.2023.1316333

**Published:** 2024-01-02

**Authors:** Aylin Kılınç Uğurlu, Ayse Özdemir Gökce, Seçil Çakır Gündoğan, Ayşe Seçil Ekşioğlu, Mehmet Boyraz

**Affiliations:** ^1^ Ankara Bilkent City Hospital, Pediatric Endocrinology Clinic, Ankara, Türkiye; ^2^ Ankara Bilkent City Hospital, Pediatric Radiology Clinic, Ankara, Türkiye

**Keywords:** early puberty -rapidly progressive, abnormal MRI findings, intracranial pathologies, incidental findings, exaggerated LH response

## Abstract

**Purpose:**

The aim of this study was to investigate the frequency and distribution of intracranial pathologies in female patients between 8 and 9 years of age who were diagnosed with early puberty (rapidly progressive) through the evaluation of MRI images.

**Materials and methods:**

A total of 74 female patients diagnosed with central precocious puberty (CPP) (6-8 years) and rapidly progressive early puberty (RPEP) (8-9 years) were included in the study. The patients were categorized into two groups, normal and abnormal, based on the findings from their MRI scans. Recent literature has classified abnormal MRI findings into three groups: pathological findings, findings with a questionable relationship to CPP, and incidental findings. Furthermore, the patients were divided into four groups based on their MRI findings and whether they had CPP or RPEP : CPP *(6-8 years)* +Normal MRI, RPEP *(8-9 years)* + Normal MRI, CPP *(6-8 years)* +Abnormal MRI, RPEP *(8-9 years)* +Abnormal MRI.

**Results:**

Out of the 74 girls included in the study, 54% (n=40) showed normal MRI results, while abnormal MRI findings were detected in 46% (n = 34) of the cases. No malignant lesions were identified among cases with abnormal MRI findings. The occurrence of abnormal MRI findings was observed in 46% of the PP group and 45% of the RPEP group. Incidental findings were the most common MRI findings in both groups. The proportion of cases with pathological findings and findings with a questionable relationship to CPP was similar in both groups (p = 0.06). Basal luteinizing hormone (LH) concentration was found to be higher in the RPEP *(8-9 years)* +Abnormal MRI group compared to the CPP *(6-8 years*) +Normal MRI group (p = 0.01).

**Conclusion:**

Our study is the first to investigate MRI findings in cases of rapidly progressive early puberty in the age range of 8–9 years. Our study demonstrates that there is no difference in terms of intracranial findings between cases of precocious puberty at the age of 6–8 years and cases of rapidly progressive early puberty aged 8-9.

## 1 Introduction

Precocious puberty refers to the early onset of puberty in girls before the age of 8 years, while early puberty is characterized by puberty onset between the ages of eight and nine (1, 2). Children with central precocious puberty (CPP) experience the onset of puberty at an early age due to the premature activation of the hypothalamic-pituitary-gonadal (HPG) axis. The use of Gonadotropin-Releasing Hormone Analog (GnRHa) therapy is a well-established and commonly employed treatment approach in the management of CPP (3). GnRH analogue treatment is also started when puberty is accelerated in cases whose puberty symptoms begin after age 8 years (2, 4). The prevalence of CNS abnormalities in girls is significantly lower compared to boys (16%-75%) (5–7), ranging from 0% to 27% across studies, and decreasing with age (8, 9). The etiology of central precocious puberty (CPP) in girls is rarely associated with organic pathologies. However, certain indications warrant cranial magnetic resonance imaging (MRI) in girls with precocious puberty, such as cases under six years of age, the presence of neurologic findings between the ages of 6-8 years, rapidly progressing puberty, and an exaggerated response to the luteinizing hormone-releasing hormone (LHRH) test (10). Despite these indications, the necessity of routine MRI scans for all females experiencing CPP between the ages of 6 and 8 years remains uncertain (11). Managing these conditions presents specific challenges in clinical practice.

The objective of this study was to investigate the frequency and distribution of intracranial pathologies in patients over eight years of age who were diagnosed with early puberty and exhibited rapid progression, using MRI imaging. By analyzing these MRI findings, we aim to enhance our understanding of the prevalence and nature of intracranial abnormalities in this population.

Understanding the presence and characteristics of intracranial pathologies in cases of early puberty-rapidly progressive is crucial for clinicians to make informed decisions regarding the need for MRI scans in this age group. By providing insights into the frequency and distribution of such pathologies, this study contributes to the ongoing discussion about the appropriateness of routine MRI screening for girls with early puberty between 8-9 aged. Ultimately, this knowledge will aid in optimizing the clinical management and care of these patients.

## 2 Materials and methods

In this retrospective study, we evaluated a cohort of 118 pediatric patients diagnosed with precocious puberty August 2019 and April 2022 at the Pediatric Endocrinology Clinic of Ankara City Hospital.

The study was performed in accordance with the Helsinki Declaration of 1975. This study was approved by the Ethics Committee of Ankara City Hospital (Approval number: E2-22-1818).

### 2.1 Definitions

*Central Precocious Puberty (CPP):* The diagnosis of precocious puberty in girls presenting with pubertal signs before 8 years of age. Diagnostic parameters included reaching Tanner stage 2 or higher, elevated basal luteinizing hormone (LH) levels greater than 0.3 IU/L, stimulated LH greater than 5 IU/L, and an LH to follicle-stimulating hormone (FSH) ratio exceeding 0.6. Radiological assessments were also considered, with an advanced bone age and pelvic ultrasound findings indicative of puberty (uterine size > 35 mm, ovarian volume > 2cc (2, 4). Additionally, the context of our demographic setting was noted, where the mean onset of puberty was 10.1 ± 1.0 years (12).

*Rapidly Progressive Puberty (RPP):* The criteria for rapidly progressive puberty, were as follows (2, 4):

• Pubertal Stage: Progression through Tanner stages within 3–6 months.
• Bone Age: Advanced bone age, at least 2 years ahead of chronological age.
• Growth Velocity: A significant annual growth velocity exceeding 6 cm.
• Predicted Adult Height*: Below target height or decreasing on serial determinations.

*Predicted Adult Height (Bayley-Pinneau Method (13)*):* A method for estimating the final adult stature of a child based on their current height, gender, and skeletal maturity. This maturity is assessed through an X-ray of the left hand and wrist to determine the bone age, which is then applied to sex-specific tables designed to forecast adult height.

*Early puberty* is characterized by puberty onset between the ages of eight and nine.

*Rapidly Progressive Early Puberty (RPEP):* RPEP was identified in those who demonstrated breast development after the age of 8 and the RPP criteria outlined above (2).

Height and weight were measured, body mass index (BMI) was calculated using the standard formula (weight in kg/(height in m^2^), and the respective standard deviation score (SDS) was calculated based on local reference data (14). Bone age was assessed by X-ray of the left hand, according to the method of Greulich and Pyle (15). FSH, LH, and estradiol (ARCHITECT System, Siemens) concentrations were measured using immunochemiluminometric assay (ICMA).

### 2.2 MRI imagining

Brain and pituitary MRIs performed to investigate the etiology were interpreted by two specialists from the Pediatric Radiology Clinic.

Cranial MRI imaging was performed for patients aged 6 to 8 years if there is rapid progression of puberty, markedly elevated basal LH or estradiol levels, or an exaggerated LH response to LHRH test according the literature (16, 17). For patients aged 8 and above Rapidly Progressive Early Puberty, MRI imaging was performed in cases due to rapid progression of puberty and also, exaggerated basal and stimulated LH responses.

Cranial MRI imaging was performed with a 1.5 Tesla General Electric Signa Explorer device. Coronal T2A, axial T2A-T1A-FLAIR images, sagittal 3D T1A Bravo, diffusion-weighted imaging and Apparent diffusion coefficient, axial-coronal-sagittal 3D T1A Bravo images after contrast were obtained for the brain. The slice thickness is 4 mm in the brain series. For the pituitary, sagittal T1A-T2A, coronal T1A-T2A images, post-contrast coronal T1A dynamic series, post-contrast sagittal, and coronal T1A images were obtained. The slice thickness of the pituitary series was 3 mm. defined pituitary enlargement and hypoplasia by comparing the mean ± SD of pituitary dimensions according to age in Turkish children (18).

### 2.3 Study groups

*Central Precocious Puberty (CPP) Group (6-8 years):* This group includes 30 patients aged between 6 and 8 years who were diagnosed with central precocious puberty. Within this age range, inclusion criteria for MR imaging encompassed patients exhibiting accelerated pubertal development or those with documented elevated basal and stimulated luteinizing hormone (LH) levels.

*Rapidly Progressive Early Puberty (RPEP) Group (8-9 years):* This group consists of 44 patients aged between 8 and 9 years who were identified with rapidly progressive early puberty. These patients showed breast development after the age of 8 and met the additional criteria for rapid progression as outlined for the rapid progression.

Excluded Group (6-8 years): 42 patients within the age range of 6 to 8 years who did not meet the criteria for rapid progression or elevated hormone levels were excluded from MRI imaging.

In our study, puberty precocious patients under the age of 6 years were not included in our study. And also, patients found to have thyroid abnormalities, adrenal dysfunction, or signs of non-classical congenital adrenal hyperplasia were excluded from the study.

The patients were divided into 2 groups, *Normal* and *Abnormal*, according to MRI findings. According to recent literature (19, 20) brain insults of Abnormal MRI findings were classified into 3 groups: *Pathological findings, Questionable relationship with CPP, and Incidental findings.*

The patients were divided into 4 groups according to MRI findings and CPP or RPEP.

•CPP *(6-8 years)* +Normal MRI
•RPEP *(8-9 years)* + Normal MRI
•CPP *(6-8 years)* +Abnormal MRI
•RPEP *(8-9 years)* +Abnormal MRI

### 2.4 Statistics analyses

All data analysis was performed with SPSS 26.0. Descriptive statistics were used to assess demographic and clinical characteristics. Data were described as a percentage and mean ± standard deviation (SD) or median (minimum-maximum) and categorical data. According to data distribution, χ2 tests, Student t-tests or Mann-Whitney U tests were used. The “Kruskal-Wallis” test was used when comparing the medians of four independent groups in the data that did not fit the normal distribution. Bonferroni correction was used in *post hoc* tests. Statistically, p<0.05 was considered significant.

## 3 Results

**Figure 1:**
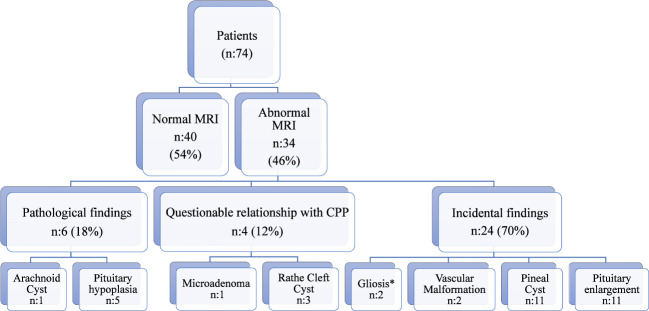
MRI findings of patients. *The two patients have also pituitary enlargement.

We conducted a retrospective analysis of 118 patients diagnosed with central precocious puberty (CPP) and rapidly progressive early puberty (RPEP). Cranial and pituitary imaging was performed in 74 of these patients, with a mean age at presentation of 8 ± 1.3 years. **Table 1** provides anthropometric findings, basal-stimulated gonadotropin and estradiol levels, bone age, and pelvic ultrasonography results for all cases. Among the analyzed cases, 54% (n=40) showed normal MRI findings, while 46% (n=34) exhibited intracranial pathologies. The abnormal MRI findings included arachnoid cyst (2%, n=1), microadenoma (2%, n=1), vascular malformation (5%, n=2), gliosis in the white matter (5%, n=2), Rathke’s cleft cyst (9%, n=3), pineal cyst (32%, n=11), pituitary enlargement (32%, n=11), and pituitary hypoplasia (14%, n=5) (**Figure 1**). Two cases showed both pituitary enlargement and gliosis. No malignant lesions were detected among the cases with abnormal MRI findings, and all neurological examinations were normal. The girl with pituitary microadenoma (dimension:3 mm) and the patients with pituitary hypoplasia underwent evaluation of various hormone levels, including LH, FSH, estradiol, prolactin, insulin-like growth factor-1 (IGF-1), thyroid function, ACTH, cortisol, as well as blood and urine osmolality. No other hormonal abnormalities were observed.

**Table 1:** Physical examination and laboratory findings of patients with normal MRI and abnormal MRI.

	Total Patients n:74	Normal MRI n:40 (54%)	Abnormal MRI n:34 (46%)	*p*-value
**Age at application (years)**	8 ± 1.3	8.1 ± 0.8	7.8 ± 1.6	0.26
**Weight (SDS)**	-0.18± -0.19	-0.2± -0.2	-0.38± -0.23	0.70
**Height (SDS)**	-0.18± -0.25	-0.2± -0.2	-0.29± -0.18	0.58
**BMI (SDS)**	0.9 ± 1.1	0.99 ± 1.03	0.80 ± 1.28	0.28
**FSH (U/L)**	5 ± 2.4	4.7 ± 2.3	5.4 ± 2.4	0.92
**LH (U/L)**	1.1 ± 1.3	0.96 ± 1.37	1.34 ± 1.37	0.12
**Estrodiol (pmol/l)**	27 ± 15	27 ± 13	27 ± 16	0.07
**Peak FSH (U/L)**	16.6 ± 9.7	14.2 ± 3.9	20 ± 14	0.20
**Peak LH (U/L)**	14.9 ± 12.1	11.4 ± 7.1	20.1 ± 15.9	0.05
**LH/FSH**	0.98 ± 0.63	0.8 ± 0.5	1.2 ± 0.7	0.15
**Uterine size (mm)**	32 ± 12	30.8 ± 14	35.2 ± 10.6	0.15
**Right ovarian volume (cc)**	3.4 ± 3.1	3.6 ± 4	3.1 ± 1.6	0.83
**Left over volume (cc)**	3 ± 2.9	3.2 ± 3.7	2.9 ± 1.4	0.70
**Bone age (years)**	9.8 ± 1.3	10 ± 0.9	9.5 ± 1.64	0.84

The values in bold represent p value < 0.05.

BA, bone age; CA, chronological age; BMI, body mass index; SDS, standard deviation score; FSH, follicle-stimulating hormone; LH, luteinizing hormone.

Comparing cases with normal and abnormal MRI findings, no significant differences were found in the mean age at diagnosis, weight (SDS), height (SDS), and BMI (SDS) (**Table 1**). Basal FSH, basal LH, peak FSH, peak LH, and LH/FSH ratios were similar between the normal MRI group and abnormal MRI group (p>0.05). Additionally, there were no differences between the groups in terms of bone age, uterine size, and ovarian volumes (p>0.05).

**Figure 2:**
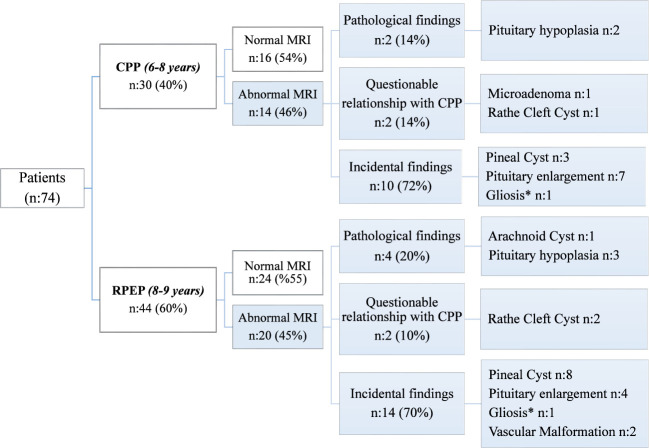
MRI findings of patients with Central Precocious Puberty (6-8 years) and Rapidly Progressive Early Puberty (8-9 years) Cases. *The two patients have also pituitary enlargement. CPP, Central Precocious Puberty; RPEP, Rapidly Progressive Early Puberty.

Abnormal MRI findings were observed in 46% of the cases in the CPP *(6-8 years)* group and 45% of the cases in the, RPEP *(8-9 years)* group. The distribution of abnormal MRI findings in these two groups, classified as pathological, with a questionable relationship to CPP, or incidental, is presented in **Figure 1**. The proportion of cases with pathological and questionable findings was similar between the groups (p=0.06). Incidental findings were the most common MRI findings in both groups. **Figure 2** presents the MRI findings for patients diagnosed with Central Precocious Puberty (ages 6–8 years) and Rapidly Progressive Early Puberty (ages 8–9 years).

**Table 2** presents the laboratory and pelvic ultrasound findings for these groups. Basal LH concentration was found to be higher in the RPEP *(8-9 years)* +Abnormal MRI group compared to the CPP *(6-8 years)* + Normal MRI group (p=0.01) (**Table 2**). In the RPEP *(8-9 years)* + Abnormal MRI group, the pathological findings included one case of arachnoid cyst, three cases of pituitary hypoplasia, and two cases of Rathke’s cleft cyst with a questionable relationship to CPP (**Figure 2**). The bone age of the CPP *(6-8 years)* + Abnormal MRI group was lower compared to the RPEP *(8-9 years)* + Normal MRI group and the RPEP *(8-9 years)* + Abnormal MRI group (p=0.001).

**Table 2:** Physical examination and laboratory findings of groups (CPP (6-8 years) +Normal MRI, RPEP (8-9 years) + Normal MRI, CPP (6-8 years) +Abnormal MRI, RPEP (8-9 years) +Abnormal MRI).

	CPP (6-8 years) Normal MRI n: 16	RPEP (8-9 years) Normal MRI n:24	CPP (6-8 years) Abnormal MRI n:14	RPEP (8-9 years) Abnormal MRI n: 20	*p*-value
**Age at application (years)**	7.5 ± 0.8	8.6 ± 0.61	7.2 ± 0.96	8.6 ± 0.36	
**FSH (U/L)**	4.7 ± 2.5	4.7 ± 2.3	5.9 ± 2.4	5.2 ± 2.4	1
**LH (U/L)**	0.46 ± 0.45	1.2 ± 1.6	0.91 ± 0.89	1.8 ± 1.6	**0.01**
**Estrodiol (pmol/l)**	22 ± 10	29 ± 15	31 ± 20	23 ± 12	0.49
**Peak FSH (U/L)**	15 ± 3.4	11.7 ± 3.6	22.7 ± 16.5	15 ± 1	0.61
**Peak LH (U/L)**	12 ± 8.8	9.8 ± 3.6	22.1 ± 17.7	18.9 ± 2.2	0.73
**LH/FSH**	0.8 ± 0.6	0.8 ± 0.3	1.29 ± 0.75	1.2 ± 0.78	0.86
**Uterine size (mm)**	30 ± 9	31 ± 16	36 ± 9.5	36 ± 11	0.85
**Right ovarian volume (cc)**	3.2 ± 1.7	3.8 ± 5	2.8 ± 1.52	3.4 ± 1.85	0.30
**Center ovarian volume (cc)**	2.6 ± 1.3	3.5 ± 4.6	2.8 ± 1.42	3 ± 1.6	0.45
**Bone age (years)**	9.5 ± 1	10.2 ± 0.97	8.4 ± 1.4	10.5 ± 1.1	**0.001**

The values in bold represent p <0.05.

CPP, Central Precocious Puberty; RPEP, Rapidly Progressive Early Puberty; BA, bone age; CA, chronological age; BMI, body mass index; SDS, standard deviation score; E2, estradiol; FSH, follicle-stimulating hormone; LH, luteinizing hormone.

## 4 Discussion

The development of precocious puberty or early puberty in children can result in premature sexual development. Magnetic resonance imaging of the brain is recommended for identifying intracranial lesions in girls with CPP. MRI imaging is indicated for patients aged 6 to 8 years if puberty progression is rapid or basal LH or estradiol is markedly elevated, or there is an exaggerated LH response to LHRH and the presence of neurological signs (16, 17). However, the routine use of MRI scans in girls with CPP remains a topic of debate, as pathological findings in girls with early puberty are limited. The necessity of MRI imaging in cases of precocious puberty has predominantly been focused on children between the ages of 6 to 8 years in the literature. However, we investigated cases that started puberty between the ages of 8-9 and had a rapid progression of puberty because there are many publications that both recommend and do not recommend MRI imaging for cases between 6-8 years with a rapid tempo of puberty, yet to the best of knowledge there was no study regarding cases between the ages of 8-9 with a rapid tempo of puberty.

We found similar rates of abnormal MRI findings in cases aged 6-8 years and those 8-9 years, with a higher prevalence of incidental findings in both groups. Notably, elevated basal luteinizing hormone (LH) concentration may serve as a warning sign for pathologic MRI findings in cases of early puberty rapid progression.

Our study’s findings, showing intracranial pathologies in 46% of CPP cases. In evaluating the necessity and outcomes of MRI screening for CPP, we must consider several pivotal studies alongside our own. A 2000 European study (21) revealed that 18.4% of girls with CPP had CNS lesions, with 8.5% being new findings, and a smaller subset involving neoplasms. a French study (22) highlighted age as a key determinant, with a higher incidence of CNS lesions (19%) in girls under 6 compared to a mere 2% incidence in those aged 6 to 8. This disparity underscores the critical nature of early onset as a factor for increased risk. In the Copenhagen study’s (11) a high frequency of pathological MRI findings, including a 0.5% rate of newly discovered tumors necessitating surgery, the authors conclude that brain MRIs should remain a standard part of evaluation for young girls with CPP. This recommendation is informed by the inability to predict pathological findings based on clinical or biochemical parameters alone. We observed that 46% of our cohort presented with intracranial pathologies. Of these cases, 70% were categorized as having incidental findings, 12% had findings of questionable relevance to central precocious puberty (CPP), and 18% demonstrated pathological changes. Noteworthy is the high incidence of pineal cysts and pituitary enlargement in our cohort, mirroring the patterns observed in the literature but raising questions about the clinical actionability of these findings. In a comprehensive review of key studies on the subject, Kaplowitz (16) concluded that routine MRI scans may not be warranted for girls aged 6–8 years with CPP, given the minimal likelihood (ranging from 0 to 2%) of identifying CNS pathologies that necessitate medical intervention. This perspective is supported by a 2019 meta-analysis by an International Consortium (3), which found that only 1.6% of girls with CPP had CNS abnormalities requiring intervention—a notably low percentage. Consequently, both Kaplowitz’s review and the Consortium’s findings underscore the importance of a balanced dialogue with parents regarding the potential benefits and limitations of MRI scanning, thereby enabling them to make well-informed decisions about their child’s care. It appears crucial to individualize the decision to perform MRIs, carefully weighing the potential for detecting actionable pathologies against the likelihood of uncovering incidental findings that may not alter clinical management. In light of this evidence, our practice aligns with current recommendations to engage in discussions with parents or guardians about the benefits and risks of MRI scanning, thereby supporting them in making informed decisions for their children with CPP.

The classification of abnormal MRI findings into pathological, questionable relationship with CPP, and incidental findings has been discussed in various studies (11, 19). This classification allows for a more comprehensive evaluation of cases with abnormal MRI findings. Consistent with the literature, our study found higher rates of incidental findings compared to pathological and questionable findings in larger case series (11). Among the incidental findings, pineal cysts were the most common in our study, consistent with previous reports (11). Pineal cysts are frequently observed in the pediatric population, with a higher prevalence in girls and older children (23). However, there is no significant correlation between pineal cysts and precocious puberty according to previous studies (24). Pituitary enlargement was the second most common incidental finding in our study, as reported in a review of 15 studies (19). Previous research has shown that pituitary volumes increase in girls with CPP compared to control groups (25, 26), and pituitary volume is associated with hormone levels (26). Beek and colleagues (27) investigated the height, length, width, sagittal cross-sectional area and volume of the pituitary gland in 12 girls (mean age 7.3 years) with central precocious puberty before and after a minimum of 6 months of treatment with GnRH analogues. They discovered no significant alterations in the size or shape of the pituitary gland in response to positive clinical outcomes of GnRH analogue treatment for central precocious puberty. The constancy of pituitary dimensions alongside the regressed puberty findings and hormonal values indicates that a hormonal increase cannot be attributed to pituitary enlargement. Yoon et al.’s (28) study delineates pituitary enlargement as an incidental finding during puberty. In their cohort, pituitary enlargement was identified in 2 (1.6%) out of 118 cases aged 6 to 6.9 years and in 9 (7%) out of 120 cases aged 7 years and older. While several research investigations have established a correlation between puberty and an increased pituitary volume, Yoon et al.’s study, together with the consistent absence of pituitary enlargement in our patient cohort, suggests that such enlargement may not be an invariable feature of normal pubertal development. But Beek et al.’s study supports the hypothesis that pituitary enlargement in the context of CPP may often be a benign, self-limiting process rather than indicative of neoplastic growth.

We identified microadenoma and Rathke’s cleft cyst in cases with a questionable relationship to CPP. In 2022, Chiu et al. (20) reported brain abnormalities in 25.3% of 251 CPP girls, they observed that pituitary microadenomas were the most prevalent brain lesions. Previous reports have shown that gonadotropin levels are similar in patients with microadenomas and those without, suggesting that these microadenomas may be non-functioning incidentalomas (20, 29). The most frequent pathological findings that lead to CPP are hypothalamic hamartomas and astrocytoma (9, 19, 30, 31). However, no tumors were detected in our study. We observed one case of an arachnoid cyst, which has been associated with CPP development in previous studies (19, 20). Pituitary hypoplasia was included among the pathological findings (9, 19, 20), but this remains a relatively unexplored topic in the literature. In their study, Fava and et al. (32) examined 112 cases of central precocious puberty (CPP) and identified pituitary hypoplasia in one patient, who also presented with deficiencies in growth and thyroid hormones. Baş et al. (33) highlighted a case of CPP development linked to a POU1F1 gene mutation, which was associated with multiple hormone deficiencies. They underscored a potential connection between the POU1F1 gene and GnRH, suggesting a genetic underpinning for the condition. Similarly, Yoon et al. (28) and Chiu et al. (20) encountered cases of CPP with pituitary hypoplasia. In our research, we evaluated patients with detected pituitary hypoplasia for anterior pituitary hormone deficiencies. These cases are monitored with anthropometric measurements and pituitary hormone levels. The relationship between CPP and pituitary hypoplasia may stem from a disruption in the balance between mediators that induce and suppress puberty along the hypothalamic-pituitary axis. However, with the current state of literature, we are unable to conclusively elucidate this mechanism.

Although basal gonadotropin levels, stimulated FSH and LH, and basal estradiol levels have been considered warning signs for intracranial malignant lesions in 6 years old cases (8, 25, 31, 32, 34), we did not detect malignant or gross intracranial pathologies in our study. The higher number of incidental findings, particularly pineal cysts, and the absence of malignant findings may contribute to this outcome. Notably, we observed that basal LH levels were higher in the RPEP group aged 8-9 years with abnormal MRIs than in the CPP group aged 6-8 years with normal MRIs. This aligns with existing literature, including Kendirci et al.’s work (25), which associates higher basal LH levels with abnormal MRI findings, suggesting that elevated basal LH should be considered a potential signal warranting. An algorithm to determine the risk of organic CPP, age below 6 years, and estradiol levels over the 45th percentile indicate a significant risk for organic brain lesion in the European population (22). In our study, there was no difference in age at diagnosis or estradiol levels between female patients with normal and abnormal MRI scans. In our study, we did not observe any differences in age at diagnosis or estradiol levels between patients with normal and abnormal MRI.

Although a comparison with the precocious puberty group under six years of age could have provided additional insights, the low number of patients in this age group in our follow-up prevented us from including them in the study. Another limitation of our study is that although the categorization of abnormal MRI results offers valuable guidance, it also introduces a degree of debate. Thus, further research is essential to validate the classification of these findings.

In conclusion, while the necessity of MR imaging for children between 6-8 years continues to be debated, our findings of similar rates of incidental, pathological, and questionable findings in both the 6-8- and 8-9-year age groups, combined with the absence of malignancy, suggest that imaging decisions must be customized on a case-by-case basis. Elevated LH levels, a consistent observation in our cohort and supported by additional studies, could be a valuable indicator for selectively determining the need for imaging in certain cases. Clinicians primarily utilize MRI to exclude the possibility of malignancy. Nevertheless, when we consider the financial costs, resource allocation, and the stress that MRI procedures can cause for children and their families, careful selection of cases is essential. The presence of similar abnormalities in the 8–9-year-old cohort with rapidly progressing puberty, a group not extensively examined in prior literature and exhibiting comparable findings to the 6–8-year age group, emphasizes the necessity to reevaluate our indications for MRI in the 6–8-year age group. Therefore, rapidly progressing puberty may not be a decisive factor in the criteria for MRI screening.

## Data availability statement

Original data generated and analyzed during this study are included in this published article or in the data repositories listed in References.

## Ethics statement

The studies involving humans were approved by The Ethics Committee of Ankara City Hospital. The studies were conducted in accordance with the local legislation and institutional requirements. Written informed consent for participation in this study was provided by the participants’ legal guardians/next of kin.

## Author contributions

AU: Conceptualization, Data curation, Formal Analysis, Investigation, Methodology, Project administration, Software, Supervision, Validation, Writing – original draft, Writing – review & editing. AG: Conceptualization, Data curation, Formal Analysis, Investigation, Methodology, Supervision, Writing – review & editing. SG: Data curation, Writing – review & editing. AE: Project administration, Writing – review & editing, Data curation, Formal Analysis, Methodology. MB: Project administration, Writing – review & editing.

## References

[1] Lanes R Soros A Jakubowicz S. Accelerated versus slowly progressive forms of puberty in girls with precocious and early puberty. Gonadotropin suppressive effect and final height obtained with two different analogs. J Pediatr Endocrinol Metab (2004) 17(5):759–66. doi: 10.1515/jpem.2004.17.5.759 15237711

[2] Howard SR de Roux N Leger J Carel J Dunkel L. Puberty and its disorders. In: Dattani MT Brook CGD, editors. Brook’s Clinical Pediatric Endocrinology, 7th ed. John Wiley & Sons (2019). p. 235–87.

[3] Bangalore K Fuqua JS Rogol D Klein KO. Use of gonadotropin-releasing hormone analogs in children : update by an international consortium. Horm Res Paediatr (2019) 91(6):357–72. doi: 10.1159/000501336 31319416

[4] Rosenfield RL Cooke DW Radovick S. Puberty in the female and its disorders. In: Sperling Pediatric Endocrinol. Elsevier (2021). p. 528–626.

[5] Wang J Zhan S Yuan J Ullah R Dong G Wu W. The incidence of brain lesions in central precocious puberty: The main cause for Chinese boys was idiopathic. Clin endocrinol (2021) 95(2):303–7. doi: 10.1111/cen.14462 PMC8362089 33721341

[6] Alikasifoglu A Vuralli D Gonc EN Ozon A Kandemir N. Changing etiological trends in male precocious puberty: evaluation of 100 cases with central precocious puberty over the last decade. Hormone Res paediatrics (2015) 83(5):340–4. doi: 10.1159/000377678 25791832

[7] Choi KH Chung SJ Kang MJ Yoon JY Lee JE Lee YA. Boys with precocious or early puberty: incidence of pathological brain magnetic resonance imaging findings and factors related to newly developed brain lesions. Ann Pediatr Endocrinol Metab (2013) 18(4):183–90. doi: 10.6065/apem.2013.18.4.183 PMC4027080 24904875

[8] Chalumeau M Chemaitilly W Trivin C Adan L Bréart G Brauner R. Central precocious puberty in girls: an evidence-based diagnosis tree to predict central nervous system abnormalities. Pediatrics (2002) 109(1):61–7. doi: 10.1542/peds.109.1.61 11773542

[9] Huynh QTV Ho BT Le NQK Trinh TH Lam LHT Nguyen NTK. Pathological brain lesions in girls with central precocious puberty at initial diagnosis in Southern Vietnam. Ann Pediatr Endocrinol Metab (2022) 27(2):105–12. doi: 10.6065/apem.2142146.073 PMC9260369 35592901

[10] Carel J-C Eugster EA Rogol A Ghizzoni L Palmert MR Group members of the E-LGACC. Consensus statement on the use of gonadotropin-releasing hormone analogs in children. Pediatrics (2009) 123(4):e752–62. doi: 10.1542/peds.2008-1783 19332438

[11] Mogensen SS Aksglaede L Mouritsen A Sørensen K Main KM Gideon P. Pathological and incidental findings on brain MRI in a single-center study of 229 consecutive girls with early or precocious puberty. PloS One (2012) 7(1):e29829. doi: 10.1371/journal.pone.0029829 22253792 PMC3257249

[12] Bundak R Darendeliler F Günöz H Baş F Saka N Neyzi O. Puberty and pubertal growth in healthy Turkish girls: no evidence for secular trend. J Clin Res Pediatr Endocrinol (2008) 1(1):8–14. doi: 10.4008/jcrpe.v1i1.16 21318059 PMC3005633

[13] Bayley N Pinneau SR. Tables for predicting adult height from skeletal age. Revised for use with the Greulich-Pyle hand standards. J Pediat (1952) 40(4):423–41. doi: 10.1016/S0022-3476(52)80205-7 14918032

[14] Neyzi O Bundak R Gökçay G Günöz H Furman A Darendeliler F. Reference values for weight, height, head circumference, and body mass index in Turkish children. J Clin Res Pediatr Endocrinol (2015) 7(4):280. doi: 10.4274/jcrpe.2183 26777039 PMC4805217

[15] Greulich WW Pyle SI. Radiographic atlas of skeletal development of the hand and wrist. The American Journal of the Medical Sciences (1959) 238(3):393.

[16] Kaplowitz PB. Do 6-8 year old girls with central precocious puberty need routine brain imaging? Int J Pediatr Endocrinol (2016) 2016(1):10–3. doi: 10.1186/s13633-016-0027-5 PMC4855709 27148371

[17] Bereket A. Santral Erken Pubertede GöRüntülemeyİ Kİmlere Yapalim? Nurçin Saka H, editor. İstanbul: Nobel Tıp Kitabevleri (2014). p. 42. DDTA.

[18] Sari S Sari E Akgun V Ozcan E Ince S Saldir M. Measures of pituitary gland and stalk: from neonate to adolescence. J Pediatr Endocrinol Metabolism : JPEM. (2014) 27(11–12):1071–6. doi: 10.1515/jpem-2014-0054 25367689

[19] Cantas-Orsdemir S Garb JL Allen HF. Prevalence of cranial MRI findings in girls with central precocious puberty: A systematic review and meta-analysis. J Pediatr Endocrinol Metab (2018) 31(7):701–10. doi: 10.1515/jpem-2018-0052 29902155

[20] Chiu CF Wang CJ Chen YP Lo FS. Pathological and incidental findings in 403 Taiwanese girls with central precocious puberty at initial diagnosis. Front Endocrinol (2020) 11(May):1–7. doi: 10.3389/fendo.2020.00256 PMC7214687 32431668

[21] Cisternino M Arrigo T Pasquino AM Tinelli C Antoniazzi F Beduschi L. Etiology and age incidence of precocious puberty in girls: a multicentric study. J Pediatr Endocrinol Metab (2000) 13(Supplement):695–702. doi: 10.1515/JPEM.2000.13.S1.695 10969911

[22] Chalumeau M Hadjiathanasiou CG Ng SM Cassio A Mul D Cisternino M. Selecting girls with precocious puberty for brain imaging: validation of European evidence-based diagnosis rule. J pediatrics (2003) 143(4):445–50. doi: 10.1067/S0022-3476(03)00328-7 14571217

[23] Al-Holou WN Garton HJL Muraszko KM Ibrahim M Maher CO. Prevalence of pineal cysts in children and young adults. J Neurosurg: Pediatrics (2009) 4(3):230–6. doi: 10.3171/2009.4.PEDS0951 19772406

[24] Çiraci S Polat R. Pineal cysts in children with precocious puberty: is it a coincidental finding? Sakarya Med J (2022) 12(2):315–21. doi: 10.31832/smj.1060348

[25] Kendirci HNP Kaba I Fidan N. Evaluatıon of Pituıtary/Cranial imagıng results of central puberty precocıous cases. Nigerian J Clin Practice (2022) 25(4):466–72. doi: 10.4103/njcp.njcp_1866_21 35439905

[26] Wu S Yang Y Wang Y Liu Q Zhu Z Gu W. Diagnostic value of pituitary volume in girls with precocious puberty. BMC Pediatr (2020) 20(1):425. doi: 10.1186/s12887-020-02283-7 32891123 PMC7487499

[27] Van Beek JT Kao SCS. Prospective assessment of pituitary size and shape on MR imaging after suppressive hormonal therapy in central precocious puberty. Pediatr Radiol (2000) 30(7):444–6. doi: 10.1007/s002470000223 10929361

[28] Yoon JS So CH Lee HS Lim JS. Prevalence of pathological brain lesions in girls with central precocious puberty : possible overestimation? J Korean Med Sci (2018) 33(51):e329. doi: 10.3346/jkms.2018.33.e329 30546283 PMC6291406

[29] Pedicelli S Alessio P Scirè G Cappa M Cianfarani S. Routine screening by brain magnetic resonance imaging is not indicated in every girl with onset of puberty between the ages of 6 and 8 years. J Clin Endocrinol Metab (2014) 99(12):4455–61. doi: 10.1210/jc.2014-2702 25238205

[30] Vurallı D Özön A Nazlı Gönç E Oğuz KK Kandemir N Alikaşifoğlu A. Gender-related differences in etiology of organic central precocious puberty. Turkish J Pediatrics (2020) 62(5):763–9. doi: 10.24953/turkjped.2020.05.007 33108078

[31] Ng SM Kumar Y Cody D Smith CS Didi M. Cranial MRI scans are indicated in all girls with central precocious puberty. Arch Dis Childhood (2003) 88(5):414–7. doi: 10.1136/adc.88.5.414 PMC1719560 12716713

[32] Fava D Calandrino A Calevo MG Allegri AEM Napoli F Gastaldi R. Clinical, endocrine and neuroimaging findings in girls with central precocious puberty. J Clin Endocrinol Metab (2022) 107(10):e4132–43. doi: 10.1210/clinem/dgac422 35881919

[33] Ba F Yava Z Toksoy G Poyrazo Ş Bundak R. Precocious or early puberty in patients with combined pituitary hormone deficiency due to POU1F1 gene mutation : case report and review of possible mechanisms. Hormones (2018) 17:581–8. doi: 10.1007/s42000-018-0079-4 30460459

[34] Vuralli D Nazli Gonc E Alikasifoglu A Kandemir N Alev Ozon Z. Central nervous system imaging in girls with central precocious puberty: When is necessary? Arch Endocrinol Metab (2020) 64(5):591–6. doi: 10.20945/2359-3997000000259 PMC10118962 34033300

